# Sex-Specific Yolk Allocation Revisited: A Test in Starlings and a Review of Evidence in Birds

**DOI:** 10.1093/iob/obag042

**Published:** 2026-07-28

**Authors:** S Casquero, A A Romero-Haro, D Gil, L Pérez-Rodríguez

**Affiliations:** Museo Nacional de Ciencias Naturales (MNCN), CSIC, c/ José Gutiérrez Abascal, 2, 28006 Madrid, Spain; Instituto de Investigación en Recursos Cinegéticos (IREC-CSIC, UCLM, JCCM), Ronda de Toledo 12, 13005 Ciudad Real, Spain; Museo Nacional de Ciencias Naturales (MNCN), CSIC, c/ José Gutiérrez Abascal, 2, 28006 Madrid, Spain; Museo Nacional de Ciencias Naturales (MNCN), CSIC, c/ José Gutiérrez Abascal, 2, 28006 Madrid, Spain; Instituto de Investigación en Recursos Cinegéticos (IREC-CSIC, UCLM, JCCM), Ronda de Toledo 12, 13005 Ciudad Real, Spain

## Abstract

Maternal effects play a key role in shaping offspring phenotype variation in birds, particularly through the allocation of different substances into the egg yolk. Among these, androgens and antioxidants are essential for early development. These compounds may exert sex-specific effects, as male and female offspring often differ in their development even from very early phases of their ontogeny. It has been therefore hypothesized that females might strategically allocate different amounts of these yolk compounds depending on the sex of the embryo. To test this hypothesis, we conducted an observational study in a wild population of spotless starlings (*Sturnus unicolor*), measured yolk concentrations of androgens (testosterone and androstenedione) and antioxidants (vitamin E, vitamin A, and carotenoids) in freshly laid eggs, and molecularly sexed the embryos from those eggs. We found no evidence that embryo sex influenced the concentration of any yolk compound. These results suggest that sex-specific allocation is constrained, possibly due to mechanistic limitations, such as the tight temporal overlap between oocyte meiosis (when embryonic sex is determined) and yolk formation. We also conducted a systematic literature review of studies that tested the association between embryo sex and yolk androgens and/or antioxidants in birds. Most studies (73%) did not report any significant effect. Among those that did, effects were often context-dependent or potentially biased by the effect of embryonic metabolism due to late sampling after laying. Overall, we found no robust evidence supporting differential maternal allocation of these yolk compounds based on embryo sex.

## Resumen

Los efectos maternos desempeñan un papel fundamental a la hora de dar forma al fenotipo de la descendencia en aves, especialmente a través de la asignación de diferentes compuestos en la yema de sus huevos. Entre estos compuestos, los andrógenos y los antioxidantes destacan por ser esenciales para el desarrollo temprano de la descendencia. Ambos grupos de compuestos pueden ejercer efectos dependientes del sexo, ya que machos y hembras difieren en sus patrones de desarrollo incluso desde las etapas más tempranas de la ontogenia. Por ello, se ha hipotetizado que las hembras podrían asignar cantidades diferentes de estos compuestos en las yemas de sus huevos según el sexo del embrión. Para poner a prueba esta hipótesis, llevamos a cabo un estudio observacional en una población silvestre de estornino negro (*Sturnus unicolor*). En huevos recién puestos, cuantificamos las concentraciones de andrógenos (testosterona y androstenediona) y antioxidantes (vitamina E, vitamina A y carotenoides) presentes en la yema y sexamos molecularmente los embriones. No encontramos evidencia de un efecto del sexo del embrión sobre la concentración de ninguno de estos compuestos de la yema. Estos resultados sugieren que la asignación diferencial según el sexo estaría limitada, posiblemente debido a restricciones inherentes a la formación del huevo, como el estrecho solapamiento temporal entre la meiosis de los ovocitos (momento en el que se determina el sexo del embrión) y la formación de la yema. Por otra parte, realizamos una revisión sistemática de la literatura sobre estudios en aves que hubieran evaluado la asociación entre el sexo del embrión y las concentraciones de andrógenos y/o antioxidantes en yema. La mayoría de los trabajos (73%) no detectaron ningún efecto significativo. Entre los que sí encontraron efectos, estos fueron dependientes del contexto o potencialmente sesgados por el metabolismo del embrión, ya que las muestras de yema se obtuvieron en fases avanzadas del desarrollo. En conjunto, no encontramos pruebas sólidas que respalden la existencia de una asignación materna diferencial de estos compuestos en la yema en función del sexo del embrión.

## Introduction

Maternal phenotype can shape offspring phenotype independently of the maternal genotype, and these changes are known as maternal effects (Mousseau and Fox 1998; Qvarnström and Price 2001). Maternal effects can be exerted from gametogenesis and embryonic development to early postnatal life, having long-lasting consequences on offspring fitness (Mousseau and Fox 1998), and can act in two different ways. On one hand, they may merely arise as side effects of the mother’s physiological state at the time of reproduction—a phenomenon called parental condition transfer effects or transmissive effects (Qvarnström and Price 2001; Marshall and Uller 2007; Crean and Bonduriansky 2014). As a consequence, offspring from mothers with a better condition will perform better than those from mothers with a poorer physiological state (i.e., silver spoon effect; Grafen 1988; Monaghan 2008). On the other hand, maternal effects may be adaptive mechanisms that have evolved to improve offspring fitness (Gluckman et al. 2005; Marshall and Uller 2007). In oviparous species as birds, these effects are particularly relevant due to the potential maternal control over egg composition. That is, the allocation of nutrients, hormones, antioxidants, and other substances into the egg can significantly shape the developmental trajectory of the embryo and its postnatal performance (Groothuis et al. 2005).

Yolk androgens are particularly relevant egg compounds due to their key role in offspring development, exerting both short-term effects during embryo stages and long-term effects throughout adulthood (Groothuis and Schwabl 2008). Indeed, they have been shown to be related to nestlings’ growth rate (Eising et al. 2001; Navara et al. 2005; Muriel et al. 2015a). Although maternal condition may influence yolk androgen allocation, the direction of this relationship remains unclear. Experimental studies where female condition was experimentally improved by supplementary feeding have yielded mixed results, including an overall reduction in yolk androgen levels (Gasparini et al. 2007), changes in patterns within the clutch (Sandell et al. 2007; Vergauwen et al. 2012), or no detectable effects (Verboven et al. 2003; Benowitz-Fredericks et al. 2013; Ruuskanen et al. 2012). Several studies have shown that maternal allocation of yolk androgens is frequently affected by the laying sequence (Schwabl 1993; Lipar and Ketterson 2000; Pilz et al. 2003; Muriel et al. 2019). Besides, environmental conditions can also affect the allocation of androgens (Benowitz-Fredericks et al. 2013), probably via their effect on maternal condition or the anticipated environment to be faced by the offspring. However, these effects have been poorly studied.

An additional type of compound that is incorporated in yolks are antioxidants: protective compounds that prevent and mitigate oxidation (Monaghan et al. 2009). Decreased or insufficient levels of antioxidants may generate an imbalance between reactive oxygen species and antioxidant capacity, leading to oxidative damage into biomolecules, compromising their functionality (i.e., oxidative stress; Monaghan et al. 2009). Yolk antioxidants are essential during embryonic development, a period characterized by a high metabolic rate due to intense physiological processes (Metcalfe and Alonso-Alvarez 2010; Smith et al. 2016), resulting in high levels of reactive oxygen species (Royle et al. 2001). Besides, the own antioxidant system is still maturing during this life period (Metcalfe and Alonso-Alvarez 2010, and references therein), making maternal-derived antioxidants essential for developing individuals. In addition, antioxidants also exert immunostimulatory functions (Surai 2002), and some of them (i.e., carotenoids) are pigments involved in sexual and parent–offspring signaling (Dugas 2009; Pérez-Rodríguez 2009; Border et al. 2023; Border and Dugas 2024). Like yolk androgens, antioxidant deposition also follows patterns according to laying sequence, usually decreasing in concentration (e.g., Royle et al. 2001; Royle et al. 2003; Török et al. 2007). Since animals cannot directly synthesize some key yolk antioxidants as vitamin E and carotenoids, these compounds must be obtained by diet (Surai 2002). There is evidence that environmental conditions can also affect the maternal deposition of these compounds (Müller et al. 2012; Benowitz-Fredericks et al. 2013).

Given the important role of yolk compounds in early development, females have been shown to adjust egg composition according to the needs of their embryos (Groothuis et al. 2005). Offspring sex is a key determinant of those developmental requirements, as males and females commonly exhibit different morphological and behavioral phenotypes (i.e., sexual dimorphism; Selander 1966; Kokko and Jennions 2008). Both sexes also differ in life-histories and trade-offs (Ball and Ketterson 2008; Tarka et al. 2018; Capilla-Lasheras et al. 2024), which likely entail distinct requirements already from the earliest stages of development (Ritchison 2023), potentially including demands for androgens and antioxidants. The contrasting responses of males and females to the manipulation of yolk components in birds indirectly support these sex-specific developmental needs. For example, in barn swallows (*Hirundo rustica*) the experimental increase of yolk androgens enhanced body mass and size of males, while reduced these traits in females (Saino et al. 2006). In the American kestrel (*Falco sparverius*), a species with reversed sexual size dimorphism since the nestling phase, the same manipulation reduced the growth rate in males, but not in females (Sockman et al. 2008). Similarly, in zebra finches (*Taeniopygia guttata*), yolk androgen increase enhanced early begging behavior in males but not in females (von Engelhardt et al. 2006). Although scarcer as compared to androgens, there is also evidence of sex-specific effects of experimental manipulations of yolk antioxidants (carotenoids) on nestling performance (Saino et al. 2011). These sex-dependent effects have fueled the idea that a differential allocation based on the (future) embryo sex could be advantageous. This idea has received considerable attention, particularly since the publication of the seminal study by Petrie and colleagues (2001), which reported remarkable differences in yolk androgen levels between eggs containing male or female embryos in the peafowl (*Pavo cristatus*). However, since then, evidence of mothers allocating yolk androgens according to embryo sex is mixed, with studies supporting (e.g., Gilbert et al. 2005; Rutstein et al. 2005; Badyaev et al. 2006) and rejecting (e.g., Eising et al. 2003; Goerlich et al. 2010; Angove et al. 2023) this possibility. Similarly, empirical studies investigating sex-specific allocation of yolk antioxidants remain scarce.

In this study, we tested the hypothesis that female birds allocate androgens and antioxidants to egg yolks differently depending on the sex of the embryo, using both an observational approach and a literature review. We conducted an observational study in a wild passerine, the spotless starling (*Sturnus unicolor*). This is a moderately polygynous and sexually dimorphic species, where adult males are slightly larger, have longer ornamental throat feathers and develop distinctive bluish and yellow beak color during the breeding season (Cordero et al. 2003). This sexual size dimorphism is present since early ages, as male nestlings are significantly larger from 5 days old (Muriel et al. 2021). Life-history strategies also differ between sexes since females can breed the first year as adults, but males usually start breeding in their second year (Redondo et al. 2022). Interestingly, experimental manipulations of yolk androgens affected early growth of male, but not female, nestlings in this species, suggesting a sex-specific sensitivity to these hormones (Muriel et al. 2013). Here, we took yolk biopsies of whole clutches at the laying date of each egg and molecularly sexed the embryos after 5 days of incubation. In these samples, we quantified levels of androstenedione and testosterone—the two main yolk androgens in avian yolks (Muriel et al. 2013)—and carotenoids, vitamin E (tocopherol), and vitamin A (retinol)—the main yolk antioxidants in birds (Surai 2002). Considering the higher growth rate of male compared to female nestlings in this species, and the sex-specific effects of yolk androgens and antioxidants in birds (see above), we predicted that, if female starlings can adjust yolk composition according to offspring sex, there should be higher concentrations of yolk androgens and antioxidants in eggs destined to produce males. Simultaneously, and to contextualize our findings in the spotless starling, we conducted a systematic review of the literature reporting differences (or lack thereof) in yolk androgens or antioxidants between eggs destined to produce male and female hatchlings.

## Methods

### Observational study in spotless starlings

#### Field work and sample collection

This observational study was carried out in a stable population of spotless starlings settled since 2003 in an open woodland near Soto del Real (Madrid, Spain). Breeding pairs in this population usually lay two clutches per season, resulting in two “clutch waves” per reproductive season (López-Rull et al. 2010; D’Arpa et al. 2021). The first clutch wave occurs in early April, when environmental conditions are optimal due to high availability of food and moderate temperatures (López-Rull et al. 2010; Salaberria et al. 2014). The second wave occurs between May and June, and conditions are harsher because of the high temperatures, lower food availability, and higher parasite loads (López-Rull et al. 2010; Salaberria et al. 2014; de la Peña et al. 2024). The modal clutch size in this population is five eggs. Starting at the end of March from the onset of nest building, we daily checked the nest boxes to detect when egg laying began and to closely monitor the egg laying sequence. We found three cases of conspecific egg parasitism in the studied clutches, identified by the occurrence of more than one egg per day. In all cases, the parasite egg was clearly distinguishable from host eggs due to its clear contrast in shape and color with the rest of the clutch, being therefore excluded from the study. We marked the eggs with a non-toxic permanent marker, weighed them with a digital balance (to the nearest 0.01 g), and measured their width and length with a digital caliper (to the nearest 0.01 mm). These measures were later used to calculate egg volume using the formula: *V*  =  0.51  ×  length  ×  width^2^ (Hoyt 1979). We performed biopsies on all eggs the day they were laid (i.e., before incubation started) to obtain an accurate measure of the yolk compounds allocated by mothers and avoiding any influence of embryonic metabolism. For biopsies, we gently cleaned a patch of the apical surface of eggs with 96° ethanol and a cotton bud and, once dried, we took one yolk biopsy per egg using sterile syringes with 30 G and 8 mm needles (Cassey et al. 2007) with the pointed side facing upward, so that the yolk would surface closely to the shell where the needles would be inserted. We made particular effort to insert the needle always to the same depth to ensure that biopsies were taken from the same region of the yolk for all eggs. We extracted between 120 and 150 mg of yolks, which were immediately split into two aliquots, one for androgens (mean ± standard deviation [SD] = 89.6 ± 35.2 mg of yolk diluted in 1 mL of ultrapure water) and other for antioxidants (40.8 ± 12.2 mg diluted in 0.2 mL of ultrapure water). The content of the biopsy was visually checked, and those containing egg white were discarded. Tubes containing the specified amount of water were previously weighed in the lab (to the nearest 0.00001 g), allowing for accurate determination of the exact dilution of both aliquots for each yolk sample. Samples were transported in a cool insulated bag to the laboratory and stored at −80°C until analysis. After biopsy, we immediately closed the egg hole with cyanoacrylate glue and, once dried, returned the eggs to the nest. Females were allowed to incubate the eggs for 5 days since clutch completion. Then, we removed complete clutches and transported all eggs to the lab in an insulated cool bag, storing them at −80°C until analysis. Differences in sample size among yolk compounds are due to assay failures or missing data.

#### Molecular sexing

We opened eggs while they were still frozen and separated embryos from yolk and albumen, taking a small portion of them for molecular sexing. With 5 days of incubation, embryos are big enough (≥0.5 cm) to allow a proper sample of them and clean them with absorbent paper to remove residual traces of surrounding compounds. We determined embryos’ sex by amplification of a CDH gene’s fragment. We used the 0057F (CGTCAATTTCCATTTCAGGTAAG) and 002R (TTATTGATCCATCAAGTCTC) primers and polymerase chain reaction (PCR) amplification was carried out in a volume 2 μl of DNA extraction (Ventim et al. 2012). PCR products were separated by electrophoresis in a 1.5% agarose gel stained with GelRed (Biotium, Fremont, California, United States of America). We used a 100-bp DNA ladder (GeneRuler, ThermoFisher, Waltham, Massachusetts, United States of America) as template. Although we biopsied 207 eggs, we were only able to determine the sex of 113 eggs from 35 clutches (79 from the first wave and 34 from the second wave), which is the actual sample size of this study.

#### Androgens assays

We quantified the concentrations of the two primary yolk androgens in this species, androstenedione and testosterone, following the protocol described in Muriel et al. (2019). We homogenized the samples using a TissueLyser II (Qiagen, Düsseldorf, Germany) and stainless-steel beads mill, and added 3 mL of mixture of petroleum and diethyl ether (30:70). The tubes were then vortexed for 15 min and centrifuged at 15,000 *g* at 4°C for 10 min. Afterward, we snapped-froze the tubes in an ethanol bath at −30°C, transferred the androgen-containing supernatant to new, clean tubes, and evaporated to dryness using nitrogen. We repeated the extraction process with the remaining pellet adding again 3 mL of the ether mixture and pooling the resulting supernatant to the previous one until evaporation. To remove protein residues, we reconstituted the dried extract with 1 mL of 90% ethanol and stored at −20°C overnight. The following day, we centrifuged tubes again at 15,000 *g* at 4°C for 10 min, and the supernatant was transferred to new tubes. We evaporated the ethanol under nitrogen and resuspended the resulting dry extract in 500 µL of steroid-free serum (DRG Labs, Germany). We used two different highly specific ELISA kits to quantify the concentrations of both androgens (androstenedione: DRG Labs, Germany; testosterone: Cayman Chemical, USA). All samples were measured in duplicate showing high within-plate repeatability (androstenedione: *r* = 0.971, standard error [SE] = 0.01, *P* < 0.001; testosterone: *r* = 0.968, SE = 0.012, *P* < 0.001).

#### Antioxidants assay

We quantified the concentrations of the main yolk carotenoids (lutein, zeaxanthin, and astaxanthin) and vitamins E and A (i.e., α-tocopherol and retinol, respectively) in this species (Casquero et al. 2025). We followed the protocol established by García-de Blas et al. (2011) and Pérez-Rodríguez et al. (2016), with some modifications (Casquero et al. 2025). We added 150 µL of pure ethanol and 150 µL of hexane: tert-butyl methyl ether (TBME) (1:1) to each tube containing a yolk sample. We homogenized the resulting mixture using stainless-steel beads mill mentioned above and added 1 mL of hexane to each sample, vortexed the tubes, and centrifuged them at 18,000 *g* at 4°C for 5 min. Then, we transferred the upper phase to new tubes. We repeated the process since the addition of hexane, transferring the resulting phase to corresponding tubes and evaporated to dryness with nitrogen. We resuspended the dry organic extract in 300 µL of MeOH:TBME:H_2_O (81:15:4). To avoid sample oxidation, we transferred the resulting extracts to chromatography amber vials and saturated them with nitrogen. Antioxidant identification and quantification was done by means of an Agilent 1200 series HPLC system (Agilent, Waldbronn, Germany) equipped with diode array (DAD) and fluorescence (FLD) detectors simultaneously, and with a C30 column (ProntoSIL 3µm 250 ×4,6 mm, Bischoff Chromatography, Leonberg, Germany) at 37°C. The volume of injections was 25 µL. We set MeOH, TBME, and ultrapure H_2_O separately and configured the device to mix the chromatography phases following these proportions to create a changing flow of phases: initially, 53% of MeOH, 45% of TBME, and 2% of H_2_O, changing in 23 min by a gradient to 10% of MeOH and 90% of TBME, and reaching finally 80% of MeOH, 16% of TBME, and 4% of H_2_O 2 min later until the end of chromatogram at 29 min. We set the flow at 1.2 mL/min during the whole procedure. Carotenoids and vitamin A were quantified with the DAD detector at 480 nm, and vitamin E with an FLD detector at 295 nm (excitation) and 325 nm (emission). We used a calibration curve that contained standards of all analyzed compounds for their quantification and identification. The repeatability of the quantification of all these antioxidants, based on a subset of nine samples extracted and quantified in duplicate was high (*r* ≥ 0.959, SE ≤ 0.041, *P* ≤ 0.001).

#### Statistical analysis

To analyze the difference in egg weight, egg volume, and yolk androgen and antioxidant concentrations between eggs destined to produce male and female offspring, we ran one linear mixed model for each response variable, including embryo sex, as well as its interaction with laying order and with the breeding event, as explanatory variables. The nest and the laboratory session were included as random factors to account for non-independence of samples among siblings and among laboratory session variations, respectively. Given that yolk lutein, zeaxanthin, and astaxanthin are strongly positively correlated in this species (Casquero et al. 2025), we used the sum of these three carotenoids (“total carotenoids,” hereafter) as response variable (results remain consistent when analyses are conducted for each carotenoid separately). Because biopsy size varied among samples, expressing compounds as ratios (e.g., ng·g^−1^ yolk) can introduce spurious associations with sample mass (Kronmal 1993; Allison et al. 1995; Mooldijk et al. 2024). To avoid this well-known issue with ratio variables, we analyzed the data using the total amount of each compound as the response variable and included yolk mass as a covariate in all models. This approach yields an equivalent interpretation to concentration values while preventing artefacts associated with mass-dependent bias in ratios. Also, yolk compounds were log-transformed to meet the assumptions of normality and homoscedasticity of residuals, which were confirmed by Shapiro Wilk tests and visual inspection. To test that our results were not affected by potential bias on sex distribution, we ran a generalized linear mixed model with a binomial distribution where sex was the binary response variable, and laying order and clutch wave the predictors, and nest a random factor to account for non-independence among eggs within the same clutch. We conducted all statistical analyses using R (v.4.3.1) (R Core Team 2021) and the package lme4 (Bates et al. 2015). We determined the significance of predictors using the Anova function of the car package (Fox and Weisberg 2019), performing F-tests for linear mixed models and likelihood ratio tests for generalized mixed models. We used the emmeans package (Lenth 2025) to perform the *post hoc* comparisons, the rptR package (Stoffel et al. 2017) to estimate the repeatability of the laboratory analyses, and the ggplot2 (Wickham 2009) and ggpubr (Kassambara 2023) packages to build the figures.

#### Literature review

We conducted a systematic search of the literature from 1993 (when the first paper about sexual differential allocation of yolk compounds was published, i.e., Schwabl [1993]) until November 2025. We performed an advanced search in Web of Science Core Collection using the following bolean terms: (androgen* OR testosterone OR androstenedione OR dihydrotestosterone OR antiox* OR carot* OR luthein OR zeaxanthin OR “vitamin A” OR “vitamin E” OR retinol OR tocopherol) and (yolk or egg) and sex* and (bird* or avian). We designed the search query to identify all studies on birds that measured yolk androgens and/or antioxidants in eggs whose embryos were sexed. From each selected study, we extracted the species examined, the living conditions (wild or captive), the yolk androgen and/or antioxidant parameters measured, the incubation day on which yolk samples were collected, and the results of the analyses testing the association between yolk compound levels and embryo sex.

## Results

### Observational study in the spotless starling

**Fig. 1:**
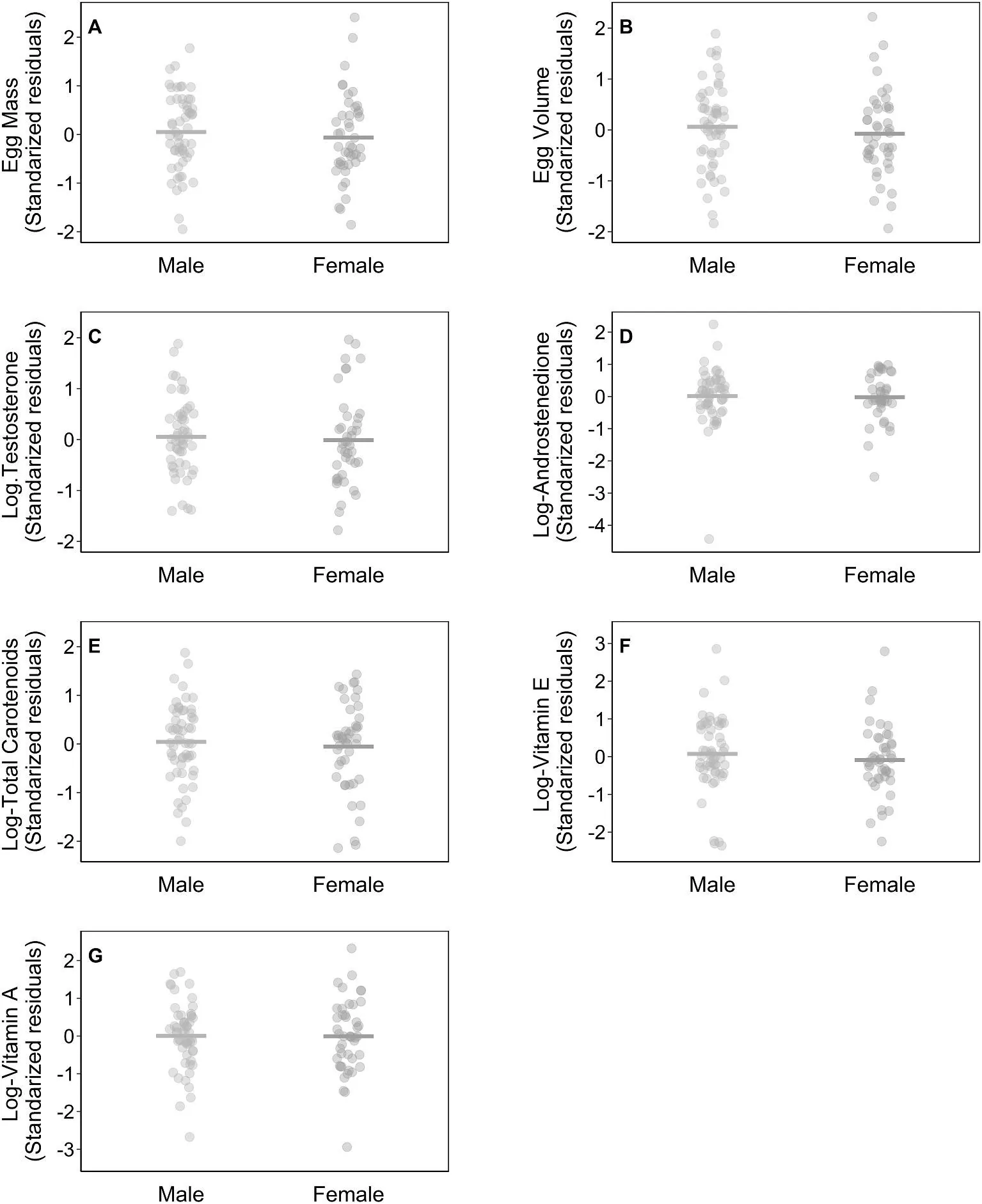
Effects of embryo sex on egg traits and yolk compounds. Dots represent individual data points and short horizontal lines indicate group means for eggs that developed into male or female embryos. Values are shown as model residuals after accounting for covariates included in the statistical models (see Methods)

Means, ranges, and SDs for all measured variables, separated by embryo sex, are shown in Table S1 of the Supplementary Material. Neither egg volume nor weight, nor the levels of androgens or any of the analyzed antioxidants (i.e., total carotenoids, vitamin E, or vitamin A) differed between eggs that developed into male or female offspring, regardless of laying order and clutch wave (Table 1 and Fig. 1). Full model results are shown in Table S2 of the Supplementary Material.

**Table 1:** Effects of embryo sex and the interaction between sex and laying order and between sex and clutch wave on the different measured egg compounds (see full models in Table S1)

	Estimate	s. e.	F	d. f.	*P*
**Egg weight (g)**					
*Sex (females)*	0.005	0.134	0.405	1, 67	0.526
Sex (females)* Laying order	−0.005	0.038	0.021	1, 67	0.886
Sex (females)* Clutch wave (2)	−0.088	0.122	0.509	1, 72	0.478
**Egg volume (cm^3^)**					
*Sex (females)*	−0.074	0.124	0.631	1, 66	0.430
Sex (females)* Laying order	0.020	0.034	0.335	1, 65	0.565
Sex (females)* Clutch wave (2)	−0.118	0.116	1.025	1, 72	0.315
**Testosterone (ng, log)**					
*Sex (females)*	0.002	0.096	0.014	1, 68.6	0.905
Sex (females)* Laying order	0.006	0.027	0.051	1, 69.6	0.822
Sex (females)* Clutch wave (2)	−0.096	0.084	1.276	1, 75.4	0.262
**Androstenedione (ng, log)**					
*Sex (females)*	−0.304	0.219	0.096	1, 59.0	0.758
Sex (females)* Laying order	0.094	0.062	2.233	1, 60.0	0.140
Sex (females)* Clutch wave (2)	−0.037	0.194	0.036	1, 63.3	0.851
**Total carotenoid (µg, log)**					
*Sex (females)*	−0.120	0.121	0.706	1, 89.4	0.403
Sex (females)* Laying order	0.028	0.034	0.652	1, 89.1	0.422
Sex (females)* Clutch wave (2)	−0.017	0.104	0.025	1, 92.0	0.874
**Vitamin E (µg, log)**					
*Sex (females)*	0.093	0.132	0.003	1, 84.3	0.954
Sex (females)* Laying order	−0.029	0.037	0.603	1, 83.2	0.440
Sex (females)* Clutch wave (2)	−0.026	0.113	0.049	1, 90.2	0.825
**Vitamin A (µg, log)**					
*Sex (females)*	−0.109	0.168	0.352	1, 73.6	0.555
Sex (females)* Laying order	0.028	0.046	0.349	1, 72.7	0.557
Sex (females)* Clutch wave (2)	−0.057	0.146	0.147	1, 82.2	0.703

### Literature review

Our literature search yielded 680 studies including the specified terms. However, only 30 studies tested the association between yolk levels of androgens and/or antioxidants and the sex of the embryo. Of these, 21 quantified only yolk androgens (Table 2), 4 quantified only yolk antioxidants (Table 3), and 5 quantified both androgens and antioxidants (Tables 2 and 3). Overall, 26 studies reported data on androgens and 9 reported data on antioxidants.

**Table 2:** Studies in birds testing for differences in yolk androgen (T: testosterone, A4: androstenedione, and DHT: dihydrotestosterone) concentrations among eggs containing male and female embryos. The condition column indicates if the study was performed in captivity (C) or in the wild (W). All yolk androgens measured in each study and the effect of embryo sex are shown. Significant effects are shown in bold. The table also indicates the days of incubation that had elapsed when the yolk samples were collected. **1:**  Müller et al. (2002); **2:**  Eising et al. (2003); **3:**  Aslam et al. (2013); **4:**  Lelono et al. (2019); **5:**  Angove et al. (2023); **6:**  Pilz et al. (2005); **7:**  Petrie et al. (2001); **8:**  Pike and Petrie (2005); **9:**  Goerlich et al. (2009); **10:**  Goerlich et al. (2010); **11:**  Rutstein et al. (2004); **12:**  Gilbert et al. (2005); **13:**  Rutstein et al. (2005); **14:**  Gilbert et al. (2007); **15:**  Pariser et al. (2012); **16:**  Schwabl (1993); **17:**  Verboven et al. (2003); **18:**  Verboven et al. (2005); **19:**  Groothuis et al. (2006); **20:**  Rubolini et al. (2011); **21:**  Partecke et al. (2020); **22:**  Lipar and Ketterson (2000); **23:**  Badyaev et al. (2006): **24:**  Gil et al. (2006); **25:**  Hegyi et al. (2011); **26:**  Isaksson et al. (2010)

Species	Condition	Parameter	Effect	Incubation	Reference
Chicken	C	T	♀ = ♂	3	1
		A4	♀ = ♂	3	
Chicken	C	T	♀ = ♂	3, 5, 7, 9, 11	2
		A4	♀ = ♂	3, 5, 7, 9, 11	
Chicken	C	T	♀ = ♂	0	3
		A4	♀ = ♂	0	
		DHT	♀ = ♂	0	
Chicken	C	T	♀ = ♂	3	4
		A4	**Wi: ♀ > ♂ but Lo: ♀ < ♂**	3	
Chicken	C	T	♀ = ♂	15, 21	5
Japanese quail	C	T	♀ = ♂	0, 10	6
		A4	♀ = ♂	0, 10	
		DHT	♀ = ♂	0, 10	
Peafowl	C	T	**♀ < ♂**	10	7
		A4	**♀ < ♂**	10	
		DHT	**♀ > ♂**	10	
Peafowl	C	T	♀ = ♂	0	8
Pigeon	C	T	♀ = ♂	3	9
Pigeon	C	T	♀ = ♂	3	10
Zebra finch	C	T + DHT	♀ = ♂	3	11
		DHT	♀ = ♂	3	
Zebra finch	C	T	**♀ > ♂**	3	12
		DHT	♀ = ♂	3	
Zebra finch	C	T + DHT	**LQ: ♀ < ♂ but HQ: ♀ = ♂**	3	13
		DHT	**LQ: ♀ < ♂ but HQ: ♀ = ♂**	3	
Zebra finch	C	T + DHT	♀ = ♂	2, 5	14
		DHT	♀ = ♂	2, 5	
		T	♀ = ♂	6	
Zebra finch	C	T + DHT	♀ = ♂	3	15
Canary	C	T	♀ = ♂	0	16
Lesser black-backed gull	W	T	♀ = ♂	4	17
		A4	♀ = ♂	4	
		DHT	♀ = ♂	4	
		Total androgens	♀ = ♂	4	
Lesser black-backed gull	W	T	♀ = ♂	4	18
Lesser black-backed gull	W	T	♀ = ♂	3	19
Yellow-legged gull	W	T	♀ = ♂	4	20
Blackbird	W	T	**Fo: ♀ = ♂ but Ci: ♀ < ♂**	4	21
		A4	♀ = ♂	4	
		DHT	♀ = ♂	4	
Red-winged blackbird	W	T	♀ = ♂	0	22
House finch	W	T	♀ = ♂	1	23
		DHT	**♀ < ♂**	1	
House martin	W	T	♀ = ♂	4	24
		A4	♀ = ♂	4	
Collared flycatcher	W	T	♀ = ♂	0	25
		A4	♀ = ♂	0	
Brown songlark	W	T	♀ = ♂	2	26
Spotless starling	W	T	♀ = ♂	0	This study
		A4	♀ = ♂	0	

**Table 3:** Studies in birds testing for differences in yolk antioxidant concentrations among eggs containing male and female embryos. The condition column indicates if the study was performed in captivity (C) or in the wild (W). All yolk antioxidants measured in each study and the effect of embryo sex are shown. Significant effects are shown in bold. The table also indicates the days of incubation that had elapsed when the yolk samples were collected. **1**: Pariser et al. (2012); **2**: Groothuis et al. (2006); **3**: Verboven et al. (2005); **4**: Bogdanova et al. (2006); **5**: Romano et al. (2008); **6**: Rubolini et al. (2011); **7**: Badyaev et al. (2006); **8**: Saino et al. (2003); **9**: Valcu et al. (2020)

Species	Condition	Parameter	Effect	Incubation	Reference
Zebra finch	C	Carotenoids + Vit E	♀ = ♂	3	1
Lesser black-backed gull	W	Carotenoids + Vit E	♀ = ♂	3	2
Lesser black-backed gull	W	Total carotenoids	**C: ♀ < ♂ but T: ♀ = ♂**	4	3
Lesser black-backed gull	W	Total carotenoids	**Y: ♀ < ♂ but M: ♀ = ♂**	4	4
Yellow-legged gull	W	Lutein	♀ = ♂	0	5
		Zeaxanthin	♀ = ♂	0	
Yellow-legged gull	W	Vitamin A	♀ = ♂	4	6
		Vitamin E	♀ = ♂	4	
		Lutein	♀ = ♂	4	
		Zeaxanthin	♀ = ♂	4	
		β-Carotene	♀ = ♂	4	
		β-Cryptoxanthin	♀ = ♂	4	
		Total carotenoids	♀ = ♂	4	
House finch	W	Vitamin A	**♀ < ♂**	1	7
		Vitamin E	♀ = ♂	1	
		Lutein	♀ = ♂	1	
		β-Carotene	**♀ > ♂**	1	
		β-Cryptoxanthin	**♀ > ♂**	1	
Barn Swallow	W	Lutein	♀ = ♂	2	8
Blue tit	W	Total carotenoids	♀ = ♂	2	9
Spotless starling	W	Vitamin A	♀ = ♂	0	This study
		Vitamin E	♀ = ♂	0	
		Total carotenoids	♀ = ♂	0	

Regarding yolk androgens, only 6 of the 26 studies (23%) reported any association between yolk androgen concentrations and embryo sex. Of these, only three studies found a direct effect of sex. Petrie et al. (2001) showed in peafowl that 10-day-incubated eggs containing a female embryo had lower levels of yolk testosterone and androstenedione, but higher levels of dihydrotestosterone than those containing a male embryo (Table 2, ref. 7). In contrast, Gilbert et al. (2005) found that 3-day-incubated eggs of zebra finches containing female embryos had higher levels of yolk testosterone than those containing male embryos, with no effects on levels of dihydrotestosterone (Table 2, ref. 12). Lastly, Badyaev et al. (2006) reported a lack of effect of embryo sex on yolk testosterone in 1-day-incubated eggs of house finches (*Haemorhous mexicanus*), but eggs containing female embryos had higher levels of dihydrotestosterone than eggs containing male embryos (Table 2, ref. 23). The remaining three studies reported a sex effect on the levels of these substances, but through interactions with other factors. Lelono et al. (2019) found that in chickens, 3-day-incubated eggs containing female embryos had higher levels of androstenedione than those containing male embryos when the father was a dominant male, but lower when the father was a subordinate male (Table 2, ref. 4). Rutstein et al. (2005) found that 3-day-incubated zebra finch eggs containing female embryos had lower levels of total androgens and dihydrotestosterone alone than eggs containing male embryos, but only when mothers were fed a low-quality diet (Table 2, ref. 13). Finally, in blackbirds (*Turdus merula*), Partecke et al. (2020) found that 4-day-incubated eggs containing a female embryo had lower levels of testosterone than eggs containing male embryos in an urban population, but not in a forest population (Table 2, ref. 21).

Regarding yolk antioxidants, three of the nine studies (33%) reported significant associations between embryo sex and antioxidant concentrations: one found a direct effect and two found interactive effects. In house finches, Badyaev et al. (2006) found that 1-day-incubated eggs containing a female embryo had lower levels of vitamin A and higher of β-carotene and β-cryptoxanthin than eggs containing a male embryo, while no differences between sexes were detected for vitamin E and lutein (Table 3, ref. 7). In lesser black-backed gulls (*Larus fuscus*), Verboven et al. (2005) found that 4-day-incubated eggs containing a female embryo had lower levels of carotenoids than eggs containing a male embryo, but this effect disappeared when maternal breeding conditions were experimentally improved (i.e., by including a breeding platform; Table 3, ref. 3). In the same species, Bogdanova et al. (2006) found that 4-day-incubated eggs containing a female embryo had lower levels of carotenoids than those containing a male embryo, but this difference was observed only in eggs from young mothers, not from old mothers (Table 3, ref. 4).

## Discussion

Here, we tested whether female starlings modulate the allocation of androgens and/or antioxidants into their eggs according to the sex of the future offspring. We found no clear evidence supporting this hypothesis, either in our observational study using the spotless starling as a model or in our literature review. These findings suggest that females may be constrained in their ability to develop such an adaptive mechanism.

Previous studies in starlings have shown that female allocation of yolk androgens depends on maternal age, social context, and environmental conditions (Pilz et al. 2003; Pilz and Smith 2004; López-Rull et al. 2010; Muriel et al. 2019). Likewise, although evidence for yolk antioxidants is scarcer, females in poor condition appear to increase yolk vitamin A levels, possibly to compensate for reduced overall egg quality (Casquero et al. 2025). Importantly, the fitness consequences of yolk androgens in spotless starlings are strongly context dependent: Elevated androgen levels enhance embryonic growth and nestling performance under favorable conditions but reduce survival and may impair immune function under harsher environments (Muriel et al. 2015b). Female starling nestlings are also more sensitive than males to adverse conditions (Gil et al. 2019), but the absence of sex differences in yolk androgens and antioxidants found here suggests that females are unable to optimize yolk composition independently for sons and daughters. This may generate a sex-related trade-off in maternal investment. If females are unable to adjust yolk composition according to embryo sex, they may instead modulate overall allocation of these compounds in relation to offspring sex ratio. Testing this possibility represents an interesting avenue for future research.

The lack of a direct association with the sex of the embryo in spotless starling eggs is consistent with most studies included in our literature review (80%, see Table 2). Most of the studies (20 out of 27) identified in our literature review analyzed androgen levels in yolk samples collected after the incubation had started (Table 2), in some cases well beyond the midpoint of the incubation period (e.g., 10 days in peafowl; Petrie et al. [2001] or 15 days in chicken; Angove et al. [2023]). Consequently, embryonic development and metabolism of yolk androgens (Wang et al. 2023) are probable causes of bias in the results. It has been often assumed that the concentrations of these compounds, when measured within 3 days of incubation, accurately reflect the levels initially allocated by the female during yolk formation (Groothuis and Von Engelhardt 2005). This assumption is often invoked for practical reasons, as at this stage of incubation the embryo is sufficiently developed to allow for the separation of embryonic tissue from the surrounding maternal vascular tissues, thereby preventing sample contamination and ensuring reliable molecular sex determination (Arnold et al. 2003; Eising et al. 2003). However, there is evidence that yolk androgens decrease from the first day of incubation, likely due—at least in partially—to embryonic metabolism (Elf and Fivizzani 2002; Winnicki et al. 2026). Consequently, studies where yolk samples were collected after the first day of incubation should not attribute observed sex differences to maternal differential deposition, but rather to embryonic metabolic processes. In addition, the neat yolk–white separation found at laying blurs away as incubation progresses, with fluid transfer between the two parts, so data on yolk concentration are impossible to obtain reliably, and whole-egg concentration must be measured. Notably, five out of the six studies that reported direct or context-dependent sex differences in yolk androgens collected yolk samples after the third day of incubation (Petrie et al. 2001; Gilbert et al. 2005; Rutstein et al. 2005; Lelono et al. 2019; Partecke et al. 2020), while the remaining study collected samples on the first day of incubation (Badyaev et al. 2006). It is also remarkable that the seminal manuscript that originated the hypothesis of a sex-dependent maternal allocation (i.e., Petrie et al. 2001) quantified yolk composition after 10 days of incubation (Table 2, ref. 7), which represents one-third of the incubation period (28–30 days) in the study species, the peafowl (Gleaves 1988). Conversely, none of the seven studies examining yolk androgens before the start of incubation (i.e., day 0) found sex effects on any of the hormones analyzed. Thus, the literature supporting the hypothesis of mothers allocating androgens according to embryo sex appears to rely on weak and potentially biased evidence, resulting from incubation-related methodological issues. Instead, it seems more plausible that early sex-specific embryonic effects, rather than maternal allocation, could explain most of the observed differences. This alternative explanation implicitly assumes that male and female avian embryos may differ in their androgen metabolism or uptake from the yolk from very early in their development. There is evidence that early incubation conditions influence androgen metabolism (Hernandez et al. 2024), and that male and female chicken embryos exhibit differential gene expression as early as 3 days after incubation begins (Liu et al. 2025), before gonadal differentiation starts. It seems unlikely that the sex of the developing embryo could meaningfully alter yolk composition before the vitelline plexus begins to develop, around stages 10–12 (ca. 36–48 h of incubation) (Hamburger and Hamilton 1951; Mueller et al. 2015). However, this potential effect would be expected to increase rapidly as incubation advances. This represents a critical point that warrants further empirical investigation.

Antioxidants have been studied far less extensively than androgens in the context of embryo-sex-related differences. In the systematic review (Table 3), we found that 3 out of 10 studies (30%) reported a direct (one study; Badyaev et al. 2006) or interactive (two studies; Verboven et al. 2005; Bogdanova et al. 2006) sex-effects. As for androgens, it has been shown that yolk antioxidants’ concentrations decrease from the onset of incubation (Winnicki et al. 2026). This may raise concerns on the interpretation of those studies where samples were collected several days after the onset of incubation (Verboven et al. 2005; Bogdanova et al. 2006). However, given the limited overall number of studies, it is still too early to draw a strong general conclusion about the existence of maternal-sex-specific antioxidant allocation.

The absence of a clear sex-specific maternal allocation, reflected both in our observational study and in the literature review, may indicate that yolk levels of these compounds are generated merely by a passive deposition, which would be mostly determined by maternal circulating levels of these compounds during yolk formation (Blount et al. 2002; López-Rull and Gil 2009), or by environmental factors determining ovarian androgen synthesis and allocation in the case of yolk androgens. However, some studies in birds like house sparrows (*Passer domesticus*) and eastern bluebirds (*Sialia sialis*) have shown how maternal plasma and yolk androgen levels are not always directly related (Mazuc et al. 2003; Navara et al. 2006b). In fact, in an experimental study in lesser black-backed gulls (*L. fuscus*), food-supplemented females showed higher plasma androgen levels but deposited less in their eggs than controls (Verboven et al. 2003). These findings suggest that yolk androgen allocation is not merely a passive consequence of maternal circulating levels. On the other hand, regarding yolk antioxidants, some studies suggest that females may be able to modulate yolk antioxidant deposition in response to external factors. For instance, house finch and zebra finch females adjusted yolk carotenoid and vitamin concentrations based on the attractiveness of their mates (Navara et al. 2006a; Williamson et al. 2006; Alonso-Alvarez et al. 2012). However, in these studies plasma antioxidant levels were not measured, so we cannot exclude the possibility that these external factors altered maternal circulating antioxidants, making it difficult to tease disentangle active from passive transfer mechanisms to yolks.

In any case, given the timing of oocyte formation and embryo sex determination, modulating yolk components allocation according to embryo sex may be more challenging than doing so in response to other factors. In birds, since females are the heterogametic sex (ZW), embryo sex is determined during oocyte meiosis, not during fertilization (Ritchison 2023). Yolk formation occurs concurrently with the release of the meiotic arrest in the oocyte and the resumption of meiosis, during which the sex chromosomes are segregated, thereby determining the genetic sex of the ovum (Ritchison 2023). Given that these processes take place simultaneously, females may just not have enough time to adjust yolk compound deposition according to the sex of the embryo. In addition to this potential mechanistic constrain, an alternative scenario has been proposed, at least for androgen deposition, in which the levels of maternal androgen would determine the outcome of meiotic division, and, consequently, the sex of the embryo (Krackow 1995; Petrie et al. 2001; Badyaev et al. 2002; Gil 2008), rather than the other way around. However, this hypothesis, which applies only to yolk androgens, leads to the same prediction tested in the current study: Eggs containing male and female embryos should differ in yolk androgen concentrations. Since neither our empirical results nor the literature review reveals strong sex differences in androgen levels, this alternative causal explanation would not be supported by our findings either.

In conclusion, the current empirical evidence (from both starlings and previous studies in other bird species) does not support the hypothesis of a differential maternal allocation of yolk androgens based on the sex of the future offspring in birds. In fact, most of the support for this hypothesis comes from data likely affected by methodological biases (Gil 2003; Groothuis et al. 2019). Similarly, evidence supporting differential maternal allocation of yolk carotenoids remains limited, largely due to the scarcity of studies. To unravel these questions, more empirical studies are demanded, specifically those analyzing yolk composition before the start of incubation. This would pave the way for the development of a meta-analytic approach, which is currently unfeasible given the limited number and heterogeneity of existing studies (e.g., quantifying different compounds with different techniques and different development status).

## Supplementary Material

obag042_Supplemental_File

## Data Availability

The data underlying this article are available in the article and in its online Supplementary Material.

## References

[bib1] Allison DB, Paultre F, Goran MI, Poehlman ET, Heymsfield SB. 1995. Statistical considerations regarding the use of ratios to adjust data. Int J Obes 19:644–52. 8574275

[bib2] Alonso-Alvarez C, Pérez-Rodríguez L, Ferrero ME, de-Blas EG, Casas F, Mougeot F. 2012. Adjustment of female reproductive investment according to male carotenoid-based ornamentation in a gallinaceous bird. Behav Ecol Sociobiol 66:731–42. 10.1007/s00265-012-1321-8

[bib3] Angove J, Willson NL, Barekatain R, Rosenzweig D, Forder R. 2023. In ovo corticosterone exposure does not influence yolk steroid hormone relative abundance or skeletal muscle development in the embryonic chicken. Poult Sci 102:102735. 10.1016/j.psj.2023.102735 37209653 PMC10208884

[bib4] Arnold KE, Orr KJ, Griffiths R. 2003. Primary sex ratios in birds: problems with molecular sex identification of undeveloped eggs. Mol Ecol 12:3451–8. 10.1046/j.1365-294X.2003.02007.x 14629359

[bib1a] Aslam MA, Hulst M, Hoving-Bolink RAH, Smits MA, de Vries B, Weites I, Groothuis TGG, Woelders H. 2013. Yolk concentrations of hormones and glucose and egg weight and egg dimensions in unincubated chicken eggs, in relation to egg sex and hen body weight. Gen Comp Endocrinol 187:15–22. 10.1016/j.ygcen.2013.02.045 23510857

[bib5] Badyaev AV, Hill GE, Whittingham LA. 2002. Population consequences of maternal effects: sex-bias in egg-laying order facilitates divergence in sexual dimorphism between bird populations. J Evol Biol 15:997–1003. 10.1046/j.1420-9101.2002.00462.x

[bib6] Badyaev AV, Seaman DA, Navara KJ, Hill GE, Mendonça MT. 2006. Evolution of sex-biased maternal effects in birds III: adjustment of ovulation order can enable sex-specific allocation of hormones, carotenoids, and vitamins. J Evol Biol 19:1044–57. 10.1111/j.1420-9101.2006.01106.x 16780506

[bib7] Ball GF, Ketterson ED. 2008. Sex differences in the response to environmental cues regulating seasonal reproduction in birds. Philos Trans R Soc, B 363:231–46. 10.1098/rstb.2007.2137 PMC2606748 17638693

[bib8] Bates D, Mächler M, Bolker BM, Walker SC. 2015. Fitting linear mixed-effects models using lme4. J Stat Softw 67:1–48. 10.18637/jss.v067.i01

[bib9] Benowitz-Fredericks ZM, Kitaysky AS, Welcker J, Hatch SA. 2013. Effects of food availability on yolk androgen deposition in the black-legged kittiwake (*Rissa tridactyla*), a seabird with facultative brood reduction. PLoS One 8:e62949. 10.1371/journal.pone.0062949 23675443 PMC3652864

[bib10] Blount JD, Surai PF, Nager RG, Houston DC, Moller AP, Trewby ML, Kennedy MW. 2002. Carotenoids and egg quality in the lesser black-backed gull *Larus fuscus*: a supplemental feeding study of maternal effects. Proc R Soc B 269:29–36. 10.1098/rspb.2001.1840 PMC1690857 11788033

[bib11] Bogdanova MI, Nager RG, Monaghan P. 2006. Does parental age affect offspring performance through differences in egg quality? Funct Ecol 20:132–41. 10.1111/j.1365-2435.2006.01088.x

[bib12] Border SE, Dugas MB. 2024. Nestling size and ornamentation interact to shape early development in house sparrow families. Biol J Linn Soc 142:410–6. 10.1093/biolinnean/blad147

[bib13] Border SE, Haas LE, Steines ME, Dugas MB. 2023. Nestling mouth colors mediate parental favoritism but do not influence detectability. Behav Ecol 34:581–92. 10.1093/beheco/arad026

[bib14] Capilla-Lasheras P, Bircher N, Brown AM, Harrison X, Reed T, York JE, Cram DL, Rutz C, Walker L, Naguib M et al. 2024. Evolution of sex differences in cooperation can be explained by trade-offs with dispersal. PLoS Biol 22:e3002859. 10.1371/journal.pbio.3002859 39446701 PMC11500963

[bib15] Casquero S, Redondo I, Gómez-Llanos E, Romero-Haro AA, Gil D, Pérez-Rodríguez L. 2025. Female starlings with experimentally impaired pre-laying condition produce smaller but vitamin a richer eggs. J Exp Zool, Part A 343:870–80. 10.1002/jez.70004 PMC12406094 40552459

[bib16] Cassey P, Ewen JG, Boulton RL, Karadas F, Moller AP, Blackburn TM. 2007. A nondestructive method for extracting maternally derived egg yolk carotenoids. J Field Ornithol 78:314–21. 10.1111/j.1557-9263.2007.00111.x

[bib17] Cordero PJ, Veiga JP, Moreno J, Parkin DT. 2003. Extra-pair paternity in the facultatively polygynous spotless starling, *Sturnus unicolor*. Behav Ecol Sociobiol 54:1–6. 10.1007/s00265-003-0603-6

[bib18] Crean AJ, Bonduriansky R. 2014. What is a paternal effect? Trends Ecol Evol 29:554–9. 10.1016/j.tree.2014.07.009 25130305

[bib19] D’Arpa SR, Muriel J, Monclús R, Gil D, Pérez-Rodríguez L. 2021. Prenatal manipulation of yolk androgen levels affects egg size but not egg colour in a songbird. Behav Ecol Sociobiol 75:52. 10.1007/s00265-021-02991-9

[bib20] de la Peña E, Muriel J, Gil D, Pérez-Rodríguez L. 2024. Parasite shedding is highly influenced by age, time of day, and sampling date in spotless starling *Sturnus unicolor* nestlings. Ardeola 71:307–20. 10.13157/arla.71.2.2024.ra6

[bib21] Dugas MB. 2009. House sparrow, *Passer domesticus*, parents preferentially feed nestlings with mouth colours that appear carotenoid-rich. Anim Behav 78:767–72. 10.1016/j.anbehav.2009.07.009

[bib22] Eising CM, Eikenaar C, Schwabl H, Groothuis TGG. 2001. Maternal androgens in black-headed gull (*Larus ridibundus*) eggs: consequences for chick development. Proc R Soc B 268:839–46. 10.1098/rspb.2001.1594 PMC1088678 11345330

[bib23] Eising CM, Müller W, Dijkstra C, Groothuis TGG. 2003. Maternal androgens in egg yolks: relation with sex, incubation time and embryonic growth. Gen Comp Endocrinol 132:241–7. 10.1016/s0016-6480(03)00090-x 12812771

[bib24] Elf PK, Fivizzani AJ. 2002. Changes in sex steroid levels in yolks of the leghorn chicken, *Gallus domesticus*, during embryonic development. J Exp Zool 293:594–600. 10.1002/jez.10169 12410608

[bib25] Fox J, Weisberg S. 2019. car: Companion to applied regression (10.32614/CRAN.package.car).

[bib2a] García-de Blas E, Mateo R, Viñuela J, Alonso-Alvarez C. 2011. Identification of carotenoid pigments and their fatty acid esters in an avian integument combining HPLC-DAD and LC-MS analyses. J Chromatogr B Biomed Appl 879:341–8. 10.1016/j.jchromb.2010.12.019 21239236

[bib3a] Gasparini J, Boulinier T, Gill VA, Gil D, Hatch SA, Roulin A. 2007. Food availability affects the maternal transfer of androgens and antibodies into eggs of a colonial seabird. J Evol Biol 20:874–80. 10.1111/j.1420-9101.2007.01315.x 17465898

[bib26] Gil D. 2003. Golden eggs: maternal manipulation of offspring phenotype by egg androgen in birds. Ardeola 50:281–94.

[bib27] Gil D. 2008. Hormones in avian eggs: physiology, ecology and behavior. In: Brockmann HJ, Roper TJ, Naguib M, WynneEdwards KE, Barnard C, Mitani JC, editors. Advances in the study of behavior. Academic Press (Elsevier), New York, United States/Oxford, United Kingdom. Vol. 38. p. 337–98. 10.1016/S0065-3454(08)00007-7

[bib28] Gil D, Alfonso-Iñiguez S, Pérez-Rodríguez L, Muriel J, Monclús R. 2019. Harsh conditions during early development influence telomere length in an altricial passerine: links with oxidative stress and corticosteroids. J Evol Biol 32:111–25. 10.1111/jeb.13396 30387533

[bib4a] Gil D, Marzal A, de Lope F, Puerta M, Moller AP. 2006. Female house martins (Delichon urbica) reduce egg androgen deposition in response to a challenge of their immune system. Behav Ecol Sociobiol 60:96–100. 10.1007/s00265-005-0145-1

[bib5a] Gilbert L, Bulmer E, Arnold KE, Graves JA. 2007. Yolk androgens and embryo sex: maternal effects or confounding factors? Horm Behav 51:231–8. 10.1016/j.yhbeh.2006.10.005 17187788

[bib29] Gilbert L, Rutstein AN, Hazon N, Graves JA. 2005. Sex-biased investment in yolk androgens depends on female quality and laying order in zebra finches (*Taeniopygia guttata*). Naturwissenschaften 92:178–81. 10.1007/s00114-004-0603-z 15668780

[bib30] Gleaves EW. 1988. G88-879 Peafowl. Historical Materials from University of Nebraska-Lincoln Extension. 1293 (https://digitalcommons.unl.edu/extensionhist/1293/).

[bib31] Gluckman PD, Hanson MA, Spencer HG. 2005. Predictive adaptive responses and human evolution. Trends Ecol Evol 20:527–33. 10.1016/j.tree.2005.08.001 16701430

[bib32] Goerlich VC, Dijkstra C, Boonekamp JJ, Groothuis TGG. 2010. Change in body mass can overrule the effects of maternal testosterone on primary offspring sex ratio of first eggs in Homing pigeons. Physiol Biochem Zool 83:490–500. 10.1086/651315 20345244

[bib6a] Goerlich VC, Dijkstra C, Schaafsma SM, Groothuis TGG. 2009. Testosterone has a long-term effect on primary sex ratio of first eggs in pigeons-in search of a mechanism. Gen Comp Endocrinol 163:184–92. 10.1016/j.ygcen.2009.01.004 19344666

[bib33] Grafen A. 1988. On the uses of data on lifetime reproductive success. Chicago: University of Chicago Press.

[bib7a] Groothuis TGG, Eising CM, Blount JD, Surai P, Apanius V, Dijkstra C, Müller W. 2006. Multiple pathways of maternal effects in black-headed gull eggs: constraint and adaptive compensatory adjustment. J Evol Biol 19:1304–13. 10.1111/j.1420-9101.2005.01072.x 16780531

[bib34] Groothuis TGG, Hsu BY, Kumar N, Tschirren B. 2019. Revisiting mechanisms and functions of prenatal hormone-mediated maternal effects using avian species as a model. Philos Trans R Soc, B 374:20180115. 10.1098/rstb.2018.0115 PMC6460091 30966885

[bib35] Groothuis TGG, Müller W, von Engelhardt N, Carere C, Eising C. 2005. Maternal hormones as a tool to adjust offspring phenotype in avian species. Neurosci Biobehav Rev 29:329–52. 10.1016/j.neubiorev.2004.12.002 15811503

[bib36] Groothuis TGG, Schwabl H. 2008. Hormone-mediated maternal effects in birds: mechanisms matter but what do we know of them? Philos Trans R Soc, B 363:1647–61. 10.1098/rstb.2007.0007 PMC2606725 18048291

[bib37] Groothuis TGG, Von Engelhardt N. 2005. Investigating maternal hormones in avian eggs: measurement, manipulation, and interpretation. In: Bauchinger U, Goymann W, JenniEiermann S editors. Bird hormones and bird migrations: analyzing hormones in droppings and egg yolks and assessing adaptations in long-distance migration. New York, United States: New York Academy of Sciences, p. 168–80. 10.1196/annals.1343.014 16055850

[bib38] Hamburger V, Hamilton HL. 1951. A series of normal stages in the development of the chick embryo. J Morphol 88:49–92. 10.1002/aja.1001950404 24539719

[bib8a] Hegyi G, Herényi M, Szöllosi E, Rosivall B, Török J, Groothuis TGG. 2011. Yolk androstenedione, but not testosterone, predicts offspring fate and reflects parental quality. Behav Ecol 22:29–38. 10.1093/beheco/arq165

[bib39] Hernandez A, Torres R, Montoya B. 2024. Incubation as a driver of maternal effects: temperature influences levels of yolk maternally derived 5α-dihydrotestosterone. Gen Comp Endocrinol 347:114420. 10.1016/j.ygcen.2023.114420 38056529

[bib40] Hoyt DF. 1979. Practical methods of estimating volume and fresh weight of bird eggs. Auk 96:73–7. 10.1093/auk/96.1.73

[bib9a] Isaksson C, Magrath MJL, Groothuis TGG, Komdeur J. 2010. Androgens during development in a bird species with extremely sexually dimorphic growth, the brown songlark, Cinclorhamphus cruralis. Gen Comp Endocrinol 165:97–103. 10.1016/j.ygcen.2009.06.015 19539628

[bib41] Kassambara A. 2023. ggpubr: ‘ggplot2’ based publication ready plots. Journal R package version 060 (https://rpkgs.datanovia.com/ggpubr/).

[bib42] Kokko H, Jennions MD. 2008. Parental investment, sexual selection and sex ratios. J Evol Biol 21:919–48. 10.1111/j.1420-9101.2008.01540.x 18462318

[bib43] Krackow S. 1995. Potential mechanisms for sex ratio adjustment in mammals and birds. Biol Rev 70:225–41. 10.1111/j.1469-185X.1995.tb01066.x 7605846

[bib44] Kronmal RA. 1993. Spurious correlation and the fallacy of the ratio standard revisited. J R Stat Soc A 156:379–92. 10.2307/2983064

[bib45] Lelono A, Riedstra B, Groothuis TGG. 2019. The relationship between male social status, ejaculate and circulating testosterone concentration and female yolk androgen transfer in red junglefowl (*Gallus gallus*). Horm Behav 116:104580. 10.1016/j.yhbeh.2019.104580 31472122

[bib46] Lenth RV. 2025. emmeans: estimated marginal means, aka least-squares means. R package version 1.10.7-100001.

[bib47] Lipar JL, Ketterson ED. 2000. Maternally derived yolk testosterone enhances the development of the hatching muscle in the red-winged blackbird *Agelaius phoeniceus*. Proc R Soc B 267:2005–10. 10.1098/rspb.2000.1242 PMC1690769 11075714

[bib48] Liu RJ, Miao HH, Zhang B, Zhang H. 2025. Identification of key differentially expressed genes during early sex determination in chicken embryos. Int J Mol Sci 26:9575. 10.3390/ijms26199575 41096840 PMC12524454

[bib49] López-Rull I, Gil D. 2009. Elevated testosterone levels affect female breeding success and yolk androgen deposition in a passerine bird. Behav Processes 82:312–8. 10.1016/j.beproc.2009.07.012 19665530

[bib50] López-Rull I, Salaberria C, Gil D. 2010. Seasonal decline in egg size and yolk androgen concentration in a double brooded passerine. Ardeola 57:321–32.

[bib51] Marshall DJ, Uller T. 2007. When is a maternal effect adaptive? Oikos 116:1957–63. 10.1111/j.2007.0030-1299.16203.x

[bib52] Mazuc J, Bonneaud C, Chastel O, Sorci G. 2003. Social environment affects female and egg testosterone levels in the house sparrow (*Passer domesticus*). Ecol Lett 6:1084–90. 10.1046/j.1461-0248.2003.00535.x

[bib53] Metcalfe NB, Alonso-Alvarez C. 2010. Oxidative stress as a life-history constraint: the role of reactive oxygen species in shaping phenotypes from conception to death. Funct Ecol 24:984–96. 10.1111/j.1365-2435.2010.01750.x

[bib54] Monaghan P. 2008. Early growth conditions, phenotypic development and environmental change. Philos Trans R Soc B 363:1635–45. 10.1098/rstb.2007.0011 PMC2606729 18048301

[bib55] Monaghan P, Metcalfe NB, Torres R. 2009. Oxidative stress as a mediator of life history trade-offs: mechanisms, measurements and interpretation. Ecol Lett 12:75–92. 10.1111/j.1461-0248.2008.01258.x 19016828

[bib56] Mooldijk SS, Labrecque JA, Ikram MA, Ikram MK. 2024. Ratios in regression analyses with causal questions. Am J Epidemiol 194:311–3. 10.1093/aje/kwae162 PMC11735960 38932568

[bib57] Mousseau TA, Fox CW. 1998. The adaptive significance of maternal effects. Trends Ecol Evol 13:403–7. 10.1016/s0169-5347(98)01472-4 21238360

[bib58] Mueller CA, Burggren WW, Tazawa H. 2015. The physiology of the avian embryo. In: Scanes CG, editor. Sturkie’s avian physiology. 6th ed. Academic Press (Elsevier), New York, United States/Oxford, United Kingdom.

[bib10a] Müller W, Eising CM, Dijkstra C, Groothuis TGG. 2002. Sex differences in yolk hormones depend on maternal social status in Leghorn chickens (Gallus gallus domesticus). Proceedings of the Royal Society B-Biological Sciences 269:2249–55. 10.1098/rspb.2002.2159 PMC1691150 12427318

[bib59] Müller W, Vergauwen J, Eens M, Blount JD. 2012. Environmental effects shape the maternal transfer of carotenoids and vitamin E to the yolk. Front Zool 9:17. 10.1186/1742-9994-9-17 PMC3502133 22876878

[bib60] Muriel J, Pérez-Rodríguez L, Gil D. 2019. Age-related patterns of yolk androgen deposition are consistent with adaptive brood reduction in spotless starlings. Behav Ecol Sociobiol 73:160. 10.1007/s00265-019-2770-0

[bib61] Muriel J, Pérez-Rodríguez L, Puerta M, Gil D. 2013. Differential effects of yolk testosterone and androstenedione in embryo development and nestling growth in the spotless starling (*Sturnus unicolor*). Gen Comp Endocrinol 194:175–82. 10.1016/j.ygcen.2013.09.013 24090611

[bib62] Muriel J, Pérez-Rodríguez L, Puerta M, Gil D. 2015a. Diverse dose-response effects of yolk androgens on embryo development and nestling growth in a wild passerine. J Exp Biol 218:2241–9. 10.1242/jeb.118257 25987739

[bib63] Muriel J, Salmón P, Nunez-Buiza A, de Salas F, Pérez-Rodríguez L, Puerta M, Gil D. 2015b. Context-dependent effects of yolk androgens on nestling growth and immune function in a multibrooded passerine. J Evol Biol 28:1476–88. 10.1111/jeb.12668 26079258

[bib64] Muriel J, Vida C, Gil D, Pérez-Rodríguez L. 2021. Ontogeny of leukocyte profiles in a wild altricial passerine. J Comp Physiol, B 191:195–206. 10.1007/s00360-020-01323-z 33196859

[bib65] Navara KJ, Badyaev AV, Mendonça MT, Hill GE. 2006a. Yolk antioxidants vary with male attractiveness and female condition in the house finch (*Carpodacus mexicanus*). Physiol Biochem Zool 79:1098–105. 10.1086/507661 17041875

[bib66] Navara KJ, Hill GE, Mendonça MT. 2005. Variable effects of yolk androgens on growth, survival, and immunity in eastern bluebird nestlings. Physiol Biochem Zool 78:570–8. 10.1086/430689 15957111

[bib67] Navara KJ, Siefferman LM, Hill GE, Mendonça MT. 2006b. Yolk androgens vary inversely to maternal androgens in eastern bluebirds: an experimental study. Funct Ecol 20:449–56. 10.1111/j.1365-2435.2006.01114.x

[bib11a] Pariser EC, Gilbert L, Hazon N, Arnold KE, Graves JA. 2012. Mind the gap: the ratio of yolk androgens and antioxidants varies between sons and daughters dependent on paternal attractiveness. Behav Ecol Sociobiol 66:519–27. 10.1007/s00265-011-1300-5

[bib68] Partecke J, Hegyi G, Fitze PS, Gasparini J, Schwabl H. 2020. Maternal effects and urbanization: variation of yolk androgens and immunoglobulin in city and forest blackbirds. Ecol Evol 10:2213–24. 10.1002/ece3.6058 32128150 PMC7042752

[bib69] Pérez-Rodríguez L. 2009. Carotenoids in evolutionary ecology: re-evaluating the antioxidant role. Bioessays 31:1116–26. 10.1002/bies.200900070 19705366

[bib12a] Pérez-Rodríguez L, García-de Blas E, Martínez-Padilla J, Mougeot F, Mateo R. 2016. Carotenoid profile and vitamins in the combs of the red grouse (Lagopus lagopus scoticus): implications for the honesty of a sexual signal. J Ornithol 157:145–53. 10.1007/s10336-015-1261-y

[bib70] Petrie M, Schwabl H, Brande-Lavridsen N, Burke T. 2001. Maternal investment—sex differences in avian yolk hormone levels. Nature 412:498–. 10.1038/35087652 11484039

[bib13a] Pike TW, Petrie M. 2005. Offspring sex ratio is related to paternal train elaboration and yolk corticosterone in peafowl. Biol Lett 1:204–7. 10.1098/rsbl.2005.0295 17148167 PMC1626203

[bib14a] Pilz KM, Adkins-Regan E, Schwabl H. 2005. No sex difference in yolk steroid concentrations of avian eggs at laying. Biol Lett 1:318–21. 10.1098/rsbl.2005.0321 17148197 PMC1617136

[bib71] Pilz KM, Smith HG. 2004. Egg yolk androgen levels increase with breeding density in the European starling, *Sturnus vulgaris*. Funct Ecol 18:58–66. 10.1111/j.1365-2435.2004.00811.x

[bib72] Pilz KM, Smith HG, Sandell MI, Schwabl H. 2003. Interfemale variation in egg yolk androgen allocation in the European starling: do high-quality females invest more? Anim Behav 65:841–50. 10.1006/anbe.2003.2094

[bib73] Qvarnström A, Price TD. 2001. Maternal effects, paternal effects and sexual selection. Trends Ecol Evol 16:95–100. 10.1016/s0169-5347(00)02063-2 11165708

[bib74] R Core Team. 2021. R: a language and environment for statistical computing (https://www.r-project.org/).

[bib75] Redondo I, Pérez-Rodríguez L, Monclús R, Muriel J, Gil D. 2022. Sexual differences in phenotypical predictors of floating status: body condition influences male but not female reproductive status in a wild passerine. Oecologia 199:79–90. 10.1007/s00442-022-05180-1 35554681 PMC9119866

[bib76] Ritchison G. 2023. In a class of their own: a detailed examination of avian forms and functions. Cham, Switzerland: Springer Nature.

[bib15a] Romano M, Caprioli M, Ambrosini R, Rubolini D, Fasola M, Saino N. 2008. Maternal allocation strategies and differential effects of yolk carotenoids on the phenotype and viability of yellow-legged gull (Larus michahellis) chicks in relation to sex and laying order. J Evol Biol 21:1626–40. 10.1111/j.1420-9101.2008.01599.x 18713240

[bib77] Royle NJ, Surai PF, Hartley IR. 2001. Maternally derived androgens and antioxidants in bird eggs: complementary but opposing effects? Behav Ecol 12:381–5. 10.1093/beheco/12.4.381

[bib78] Royle NJ, Surai PF, Hartley IR. 2003. The effect of variation in dietary intake on maternal deposition of antioxidants in zebra finch eggs. Funct Ecol 17:472–81. 10.1046/j.1365-2435.2003.00752.x

[bib16a] Rubolini D, Romano M, Navara KJ, Karadas F, Ambrosini R, Caprioli M, Saino N. 2011. Maternal effects mediated by egg quality in the Yellow-legged Gull Larus michahellis in relation to laying order and embryo sex. Front Zool 8. 10.1186/1742-9994-8-24. PMC3214788 22011400

[bib17a] Rutstein AN, Gilbert L, Slater PJB, Graves JA. 2004. Mate attractiveness and primary resource allocation in the zebra finch. Anim Behav 68:1087–94. 10.1016/j.anbehav.2004.02.011

[bib79] Rutstein AN, Gilbert L, Slater PJB, Graves JA. 2005. Sex-specific patterns of yolk androgen allocation depend on maternal diet in the zebra finch. Behav Ecol 16:62–9. 10.1093/beheco/arh123

[bib18a] Ruuskanen S, Doligez B, Pitala N, Gustafsson L, Laaksonen T. 2012. Long-term fitness consequences of high yolk androgen levels: sons pay the costs. Funct Ecol 26:884–94. 10.1111/j.1365-2435.2012.01994.x

[bib80] Saino N, Ferrari RP, Romano M, Martinelli R, Lacroix A, Gil D, Moller AP. 2006. Maternal allocation of androgens and antagonistic effects of yolk androgens on sons and daughters. Behav Ecol 17:172–81. 10.1093/beheco/arj023

[bib81] Saino N, Romano M, Caprioli M, Rubolini D, Ambrosini R. 2011. Yolk carotenoids have sex-dependent effects on redox status and influence the resolution of growth trade-offs in yellow-legged gull chicks. Behav Ecol 22:411–21. 10.1093/beheco/arq220

[bib19a] Saino N, Romano M, Ferrari RP, Martinelli R, MØller AP. 2003. Maternal antibodies but not carotenoids in barn swallow eggs covary with embryo sex. J Evol Biol 16:516–22. 10.1046/j.1420-9101.2003.00534.x 14635852

[bib82] Salaberria C, Celis P, López-Rull I, Gil D. 2014. Effects of temperature and nest heat exposure on nestling growth, dehydration and survival in a Mediterranean hole-nesting passerine. Ibis 156:265–75. 10.1111/ibi.12121

[bib20a] Sandell MI, Adkins-Regan E, Ketterson ED. 2007. Pre-breeding diet affects the allocation of yolk hormones in zebra finches Taeniopygia guttata. J Avian Biol 38:284–90. 10.1111/j.2007.0908-8857.03640.x

[bib83] Schwabl H. 1993. Yolk is a source of maternal testosterone for developing birds. Proc Natl Acad Sci USA 90:11446–50. 10.1073/pnas.90.24.11446 8265571 PMC48000

[bib84] Selander RK. 1966. Sexual dimorphism and differential niche utilization in birds. Condor 68:113–51. 10.2307/1365712

[bib85] Smith SM, Nager RG, Costantini D. 2016. Meta-analysis indicates that oxidative stress is both a constraint on and a cost of growth. Ecol Evol 6:2833–42. 10.1002/ece3.2080 27217942 PMC4863009

[bib86] Sockman KW, Weiss J, Webster MS, Talbott V, Schwabl H. 2008. Sex-specific effects of yolk-androgens on growth of nestling American kestrels. Behav Ecol Sociobiol 62:617–25. 10.1007/s00265-007-0486-z

[bib87] Stoffel MA, Nakagawa S, Schielzeth H. 2017. rptR: repeatability estimation and variance decomposition by generalized linear mixed-effects models. Methods Ecol Evol 8:1639–44. 10.1111/2041-210x.12797

[bib88] Surai PF. 2002. Natural antioxidants in avian nutrition and reproduction. Nottingham, United Kingdom: Nottingham University Press.

[bib89] Tarka M, Guenther A, Niemelä PT, Nakagawa S, Noble DWA. 2018. Sex differences in life history, behavior, and physiology along a slow-fast continuum: a meta-analysis. Behav Ecol Sociobiol 72:132. 10.1007/s00265-018-2534-2 30100667 PMC6060830

[bib90] Török J, Hargitai R, Hegyi G, Matus Z, Michl G, Péczely P, Rosivall B, Tóth G. 2007. Carotenoids in the egg yolks of collared flycatchers (*Ficedula albicollis*) in relation to parental quality, environmental factors and laying order. Behav Ecol Sociobiol 61:541–50. 10.1007/s00265-006-0282-1

[bib21a] Valcu CM, Valcu M, Teltscher K, Kempenaers B. 2020. Non-analytical methods for the estimation of total yolk carotenoids in passerine eggs. Ibis 162:1075–81. 10.1111/ibi.12803

[bib91] Ventim R, Morais J, Pardal S, Mendes L, Ramos JA, Pérez-Tris J. 2012. Host-parasite associations and host-specificity in haemoparasites of reed bed passerines. Parasitology 139:310–6. 10.1017/s0031182011002083 22217333

[bib92] Verboven N, Evans NP, D’Alba L, Nager RG, Blount JD, Surai PF, Monaghan P. 2005. Intra-specific interactions influence egg composition in the lesser black-backed gull (*Larus fuscus*). Behav Ecol Sociobiol 57:357–65. 10.1007/s00265-004-0862-x

[bib93] Verboven N, Monaghan P, Evans DM, Schwabl H, Evans N, Whitelaw C, Nager RG. 2003. Maternal condition, yolk androgens and offspring performance: a supplemental feeding experiment in the lesser black-backed gull (*Larus fuscus*). Proc R Soc B 270:2223–32. 10.1098/rspb.2003.2496 PMC1691499 14613608

[bib22a] Vergauwen J, Goerlich VC, Groothuis TGG, Eens M, Müller W. 2012. Food conditions affect yolk testosterone deposition but not incubation attendance. Gen Comp Endocrinol 176:112–9. 10.1016/j.ygcen.2012.01.003 22265816

[bib94] von Engelhardt N, Carere C, Dijkstra C, Groothuis TGG. 2006. Sex-specific effects of yolk testosterone on survival, begging and growth of zebra finches. Proc R Soc B 273:65–70. 10.1098/rspb.2005.3274 PMC1560008 16519236

[bib95] Wang YQ, Riedstra B, de Vries B, van Faassen M, Pranger A, Kema I, Groothuis T. 2023. Plasticity in metabolism of maternal androgens in avian embryos. Sci Rep 13:18255. 10.1038/s41598-023-35340-z PMC10195863 37202471

[bib96] Wickham H. 2009. ggplot2: elegant graphics for data analysis (10.1007/978-0-387-98141-3).

[bib97] Williamson KA, Surai PF, Graves JA. 2006. Yolk antioxidants and mate attractiveness in the Zebra Finch. Funct Ecol 20:354–9. 10.1111/j.1365-2435.2006.01087.x

[bib98] Winnicki SK, Benson TJ, Paitz RT, McGraw KJ, Hauber ME. 2026. Laying order predicts avian maternal investment in egg yolk hormones and nutrients but not embryo size in the asynchronously hatching *Turdus migratorius* (American robin). Ornithology published online (10.1093/ornithology/ukag018).

