# Supplementary material for: Sex-Specific Yolk Allocation Revisited: A Test in Starlings and a Review of Evidence in Birds
Source: Integr Org Biol. 2026 Jul 28;8(1):obag042. doi: 10.1093/iob/obag042 (PMC13539270; doi:10.1093/iob/obag042)
Supplement: Supplementary file 1 [file obag042_supplemental_file.docx]

**Supplementary Material**

**Sex-specific yolk allocation revisited: a test in starlings and a review of evidence in birds**

Casquero, S.^1,2^, Romero-Haro, A. A.^1^, Gil, D.^1^, Pérez-Rodríguez, L.^2^

^1^ Museo Nacional de Ciencias Naturales (MNCN), CSIC, c/ José Gutiérrez Abascal, 2, 28006 Madrid, Spain

^2^ Instituto de Investigación en Recursos Cinegéticos (IREC), CSIC-UCLM-JCCM, Ronda de Toledo 12, 13005 Ciudad Real, Spain

*corresponding: silvia.casquero@mncn.csic.es

ORCID: Silvia Casquero: <https://orcid.org/0000-0002-5466-7770>; Ana Ángela Romero-Haro: <https://orcid.org/0000-0002-7127-4733>; Diego Gil: <https://orcid.org/0000-0003-3179-1987>; Lorenzo Pérez-Rodríguez: <https://orcid.org/0000-0002-5926-1438>.

**Table S1** Egg weight, volume, and yolk androgen and antioxidant concentrations (means, standard deviations, and ranges) in spotless starling eggs that developed into male or female embryos.

|  | **Male Embryo** | | | **Female Embryo** | | |
| --- | --- | --- | --- | --- | --- | --- |
|  | **Mean** | **s. d.** | **Range** | **Mean** | **s. d.** | **Range** |
| **Egg weight (g)** | 7.45 | 0.49 | 6.25 - 8.37 | 7.42 | 0.53 | 6.45 - 9.02 |
| **Egg volume (cm^3^)** | 7.10 | 0.48 | 5.92 - 8.02 | 7.07 | 0.50 | 6.03 - 8.52 |
| **Testosterone Levels (ng/g)** | 2.09 | 0.59 | 1.19 - 4.52 | 1.90 | 0.62 | 0.92 - 4.50 |
| **Androstenedione Levels (ng/g)** | 3.49 | 2.07 | 1.05 - 9.90 | 3.75 | 2.56 | 1.15 - 11.2 |
| **Total Carotenoid Levels (µg/g)** | 101 | 49.2 | 35.1 - 212 | 91.4 | 46.7 | 14.6 - 186 |
| **Vitamin E Levels (µg/g)** | 29.0 | 10.9 | 9.38 - 65.9 | 27.1 | 10.4 | 11.0 - 65.3 |
| **Vitamin A Levels (µg/g)** | 2.49 | 0.67 | 1.30 - 3.93 | 2.72 | 0.94 | 0.74 - 4.85 |

**Table S2** Full models testing for the effects of embryo sex, laying order, clutch wave and the interaction between sex and laying order and between sex and clutch wave on the different measured egg compounds

|  | **Estimate** | **s. e.** | **F** | **d.f.** | **P** |
| --- | --- | --- | --- | --- | --- |
| **Egg weight (g)** |  |  |  |  |  |
| *Sex (females)* | 0.005 | 0.134 | 0.405 | 1, 67.0 | 0.526 |
| *Laying order* | -0.054 | 0.025 | 10.40 | 1, 66.1 | 0.002 |
| *Clutch wave (2)* | 0.069 | 0.174 | 0.034 | 1, 33.3 | 0.855 |
| *Sex (females)* Laying order* | -0.005 | 0.038 | 0.021 | 1, 66.6 | 0.886 |
| *Sex (females)* Clutch wave (2)* | -0.088 | 0.122 | 0.509 | 1, 72.2 | 0.478 |
| **Egg volume (cm^3^)** |  |  |  |  |  |
| *Sex (females)* | -0.074 | 0.124 | 0.631 | 1, 65.7 | 0.430 |
| *Laying order* | -0.054 | 0.023 | 7.691 | 1, 64.3 | 0.007 |
| *Clutch wave (2)* | 0.130 | 0.172 | 0.212 | 1, 33.3 | 0.649 |
| *Sex (females)* Laying order* | 0.020 | 0.034 | 0.335 | 1, 65.0 | 0.565 |
| *Sex (females)* Clutch wave (2)* | -0.118 | 0.116 | 1.025 | 1, 71.7 | 0.315 |
| **Testosterone (ng, log)** |  |  |  |  |  |
| *Sex (females)* | 0.002 | 0.096 | 0.014 | 1, 68.6 | 0.905 |
| *Laying order* | 0.021 | 0.018 | 2.813 | 1, 70.7 | 0.098 |
| *Clutch wave (2)* | 0.372 | 0.101 | 12.20 | 1, 61.7 | 0.001 |
| *Yolk sample weight* | 0.003 | 0.001 | 8.295 | 1, 75.8 | 0.005 |
| *Sex (females)* Laying order* | 0.006 | 0.027 | 0.051 | 1, 69.6 | 0.822 |
| *Sex (females)* Clutch wave (2)* | -0.096 | 0.084 | 1.276 | 1, 75.4 | 0.262 |
| **Androstenedione (ng, log)** | |  |  |  |  |
| *Sex (females)* | -0.304 | 0.219 | 0.096 | 1, 59.0 | 0.758 |
| *Laying order* | 0.054 | 0.042 | 8.116 | 1, 60.2 | 0.006 |
| *Clutch wave (2)* | 0.261 | 0.258 | 1.000 | 1, 53.7 | 0.322 |
| *Yolk sample weight* | 0.009 | 0.003 | 11.08 | 1, 63.5 | 0.001 |
| *Sex (females)* Laying order* | 0.094 | 0.062 | 2.233 | 1, 60.0 | 0.140 |
| *Sex (females)* Clutch wave (2)* | -0.037 | 0.194 | 0.036 | 1, 63.3 | 0.851 |
| **Total Carotenoid (µg, log)** | |  |  |  |  |
| *Sex (females)* | -0.120 | 0.121 | 0.706 | 1, 89.4 | 0.403 |
| *Laying order* | -0.104 | 0.024 | 23.37 | 1, 84.5 | 0.000 |
| *Clutch wave (2)* | -0.054 | 0.080 | 1.109 | 1, 35.2 | 0.299 |
| *Yolk sample weight* | 0.010 | 0.002 | 18.73 | 1, 88.7 | 0.000 |
| *Sex (females)* Laying order* | 0.028 | 0.034 | 0.652 | 1, 89.1 | 0.422 |
| *Sex (females)* Clutch wave (2)* | -0.017 | 0.104 | 0.025 | 1, 92.0 | 0.874 |
| **Vitamin E (µg, log)** |  |  |  |  |  |
| *Sex* | 0.093 | 0.132 | 0.003 | 1, 84.3 | 0.954 |
| *Laying order* | 0.030 | 0.026 | 0.753 | 1, 80.1 | 0.388 |
| *Clutch wave (2)* | 0.233 | 0.094 | 8.228 | 1, 35.7 | 0.007 |
| *Yolk sample weight* | 0.015 | 0.002 | 34.65 | 1, 86.0 | 0.000 |
| *Sex (females)* Laying order* | -0.029 | 0.037 | 0.603 | 1, 83.2 | 0.440 |
| *Sex (females)* Clutch wave (2)* | -0.026 | 0.113 | 0.049 | 1, 90.2 | 0.825 |
| **Vitamin A (µg, log)** |  |  |  |  |  |
| *Sex* | -0.109 | 0.168 | 0.352 | 1, 73.6 | 0.555 |
| *Laying order* | -0.206 | 0.032 | 59.17 | 1, 73.5 | 0.000 |
| *Clutch wave (2)* | 0.279 | 0.150 | 3.468 | 1, 35.1 | 0.071 |
| *Yolk sample weight* | 0.020 | 0.003 | 37.86 | 1, 77.0 | 0.000 |
| *Sex (females)* Laying order* | 0.028 | 0.046 | 0.349 | 1, 72.7 | 0.557 |
| *Sex (females)* Clutch wave (2)* | -0.057 | 0.146 | 0.147 | 1, 82.2 | 0.703 |
